# Optimal Design of pH-neutral Geopolymer Foams for Their Use in Ecological Plant Cultivation Systems

**DOI:** 10.3390/ma12182999

**Published:** 2019-09-16

**Authors:** Magdalena Szechyńska-Hebda, Joanna Marczyk, Celina Ziejewska, Natalia Hordyńska, Janusz Mikuła, Marek Hebda

**Affiliations:** 1 The Franciszek Górski Institute of Plant Physiology Polish Academy of Sciences, Niezapominajek 21, 30-239 Cracow, Poland; n.hordynska@ifr-pan.edu.pl; 2 The Plant Breeding and Acclimatization Institute - National Research Institute, Radzików, 05-870 Błonie, Poland; 3 Institute of Materials Engineering, Cracow University of Technology, Warszawska 24, 31-155 Cracow, Poland; jmarczyk94@gmail.com (J.M.); celina.ziejewska@gmail.com (C.Z.); jamikula@pk.edu.pl (J.M.)

**Keywords:** cellulose fibres, geopolymer foam, neutralisation, plant cultivation

## Abstract

We have calculated that with the world population projected to increase from 7.5 billion in 2017 to 9.8 in 2050, the next generation (within 33 years) will produce 12,000–13,000 Mt of plastic, and that the yearly consumption will reach 37–40 kilos of plastic per person worldwide. One of the branches of the plastics industry is the production of plastics for agriculture e.g., seed trays and pots. In this paper, novel metakaolin-based geopolymer composites reinforced with cellulosic fibres are presented as an alternative to plastic pots. Materials can be dedicated to agricultural applications, provided they have neutral properties, however, geopolymer paste and its final products have high pH. Therefore, a two-step protocol of neutralisation of the geopolymer foam pots was optimised and implemented. The strength of the geopolymer samples was lower when foams were neutralised. The reinforcement of geopolymers with cellulose clearly prevented the reduction of mechanical properties after neutralisation, which was correlated with the lower volume of pores in the foam and with the cellulose chemical properties. Both, neutralisation and reinforcement with cellulose can also eliminate an efflorescence. Significantly increased plant growth was found in geopolymer pots in comparison to plastic pots. The cellulose in geopolymers resulted in better adsorption and slower desorption of minerals during fertilisation. This effect could also be associated with a lower number of large pores in the presence of cellulose fibres in pots, and thus more stable pore filling and better protection of internal surface interactions.

## 1. Introduction

The exponential increase of the world’s population and production intensity causes a lot of environmental problems, amongst them, an unparalleled volume of generated waste [1,2]. The pollution of natural ecosystems with waste plastics is one of the most massive and urgent problems. It is estimated that the current global production of plastic reaches 350–380 Mt with a compound annual growth rate of 8.4% [3]. The total economic damage to the world’s ecosystems caused by plastic, amounts to at least $13 billion every year [4]. We have calculated that, the next generation will produce 12,000–13,000 Mt of plastic, and the yearly consumption will reach 37–40 kilos of plastic per world citizen. In order to limit the harmful effects of plastic production on the environment, and in turn human health, appropriate waste management is necessary. On 24 October 2018, members of the European Parliament voted overwhelmingly in favour of proposals from the European Commission to cut plastics waste, and proposed the new ‘Single-Use Plastics Directive’.

One of the branches of the plastics industry is the production of plastics for agriculture and horticulture e.g., seed trays and pots that are a staple in most greenhouse cultivations. A small sized company can process 10,000 tonnes of plastic per year for a total production of about 20–50 million pots, and it is estimated that around 500–800 million plastic plant pots are used every year in each European country. Many pots are simply discarded after plants have been planted in the ground. Considering all of the above data, biodegradable alternatives to plastic plant pots are a challenge for producers. Fibrous bio-based materials such as coir, manure, peat, rice hull, straw and wood pulp, or even recycled paper can be pressed into the shape of a plant pot. However, most of these alternative containers for growing plants can easily be damaged, and in some cases more resistant materials are required for outdoor planting. 

As an effect of fires in France between 1970–1973, which involved common organic plastics, the interest in alternative non-flammable materials has increased [5], and has directly led to the development of geopolymers. Due to excellent resistance, low shrinkage and creep abilities, high compressive strength and durability [6,7], geopolymers are being examined in many industrial, and scientific disciplines [8,9,10,11]. They are used as cements and concretes [12,13], heat-resistant materials [14,15], high-tech composites [16], anti-microbial materials for medical applications [17], adsorbents/ion-exchangers, photocatalysts, high-pressure membranes, filter media, materials, pH buffers, carrier media in bioreactors, and materials allowing solidification/stabilisation of dyes and heavy metals for applications in water and wastewater treatment [18,19,20]. However, despite all of this they are not considered as an alternative to plastics.

The term ‘‘geopolymer’’ [21] was introduced to describe the amorphous inorganic aluminosilicate material produced by reacting Si- and Al-rich metakaolin [13], or industrial by-products (fly ash, clay, blast furnace slag) with an alkaline aqueous solution [22,23,24,25]. Geopolymers are produced by the following reactions: (1) Dissolution of metakaolin into silicate monomers and aluminate monomers; (2) polymerisation of monomers into aluminosilicate oligomers, and then into small geopolymer fragments or ’proto-zeolitic nuclei’ (thermodynamically metastable and incompletely cross-linked); (3) fragments combination into larger molecules, that finally form aluminosilicate inorganic polymer gels, and crystallised phases, consisting of SiO_4_ and AlO_4_ tetrahedra sharing oxygen corners. If foaming agents (hydrogen peroxide, sodium perborate, metallic Al or Si powder) are added into the geopolymer paste during its consolidation, lightweight, and resistant geopolymer foams with pore sizes ranging from nanometers to a few millimetres, and a total porosity of up to 90% can be obtained [26,27,28,29,30,31]. However, despite their many desirable attributes, the cellular structure of geopolymer foams may lead to an inferior mechanical performance e.g., increased product fragility. This limitation may be overcome by reinforcing the matrix with carbon, and glass fibres, coated steel fibres [32,33], polyethylene, and polyvinyl alcohol fibres [34]. Natural wool and different plant-originated fibres (hemp, kenaf, cotton, sheep wool, coconut) have also been used to develop innovative geopolymer systems for building retrofitting and improvement of energy efficiency [35,36,37,38]. Natural fibres used as reinforcement in brittle inorganic matrices can significantly improve mechanical properties in terms of fracture toughness, flexural and impact strength [39,40,41,42,43], and reduce cost as well as have a positive impact in terms of the environment. 

In this paper, for the first time, we report on the preparation and characterisation of new metakaolin-based geopolymer composites, reinforced with cellulosic fibres, which are dedicated to agricultural applications. The foamed geopolymer material with pH close to 7 can be easily formed as cultivation pots. Furthermore, such pots, soaked with a water solution of nutrients, provide a prolonged and gradual dosage release of fertiliser during plant cultivation.

## 2. Materials and Methods

### 2.1. Materials

Metakaolin KM 60 (Keramost) with chemical composition: 50–55% SiO_2_, min. 40% Al_2_O_3_, max. 1.45% Fe_2_O_3_, 0.05–0.5% CaO, 0.20–0.45% MgO, max. 1.5% K_2_O + Na_2_; and quartz sand with chemical composition: 90.0–90.3% SiO_2_, max. 0.2% Fe_2_O_3_, 0.08–0.1% TiO_2_, 0.4–0.7% Al_2_O_3_, 0.17% CaO, 0.01% MgO, were used as base materials (in proportion 9:1). The process of alkaline activation was carried out using an aqueous solution of sodium silicate (R-145) with a molar module of 2.5, mixed up with an 8M NaOH solution in a ratio of 2.5:1. The ratio of metakaolin/sand to alkali activators was in the range 0.59 to 0.84 (Table 1). In order to obtain foamed geopolymers, 99.7% purity aluminium powder (Fluca) with particle size in the range 10–200 μm, and 3.5% hydrogen peroxide solution (Sigma-Aldrich) were added. Geopolymers were reinforced with cellulose ProMC^2000^ (ProAgro) with the length of cellulose fibres being 1350 μm, 50–70 g/L apparent density, and pH 7.0–8.0. The chemical composition of the mineral solution added to the geopolymer paste, or used to soak the formed geopolymer pots, consisted of: 5% N, 4% P, 6% K, 0.02% B, 0.002% Cu, 0.02% Fe, 0.015% Mn, 0.002% Mo and 0.015% Zn.

### 2.2. Geopolymer Foam Design

The geopolymer materials were manufactured according to different formulations, in which two components (mineral solution and cellulose fibres) were added to produce four different combinations, and to investigate the influence of these additions on the geopolymerisation process, as well as the final properties of the materials. The basic procedure for production of geopolymer foam pots (G) involved: (1) Mixing sodium hydroxide solution with the sodium silicate solution (water glass); (2) mixing metakaolin, quartz sand, and alkaline solution in a cement mortar mixer (GEOLAB) for 10 min at 800 rpm to form a homogeneous paste; (3) adding hydrogen peroxide and aluminium powder to produce a geopolymer foam material. The methodology of producing geopolymer foam pots with the addition of mineral solution (GM) proceeded in the same way as for the base geopolymers. The modification consisted of the introduction of the fertiliser at the stage of mixing the ingredients with the alkaline solution. During the production of organic-geopolymer hybrid foams (GC and GCM), cellulose fibres were added to the mixture of metakaolin and sand. The percentage of individual components for all geopolymer materials was calculated so as to maintain a constant liquid to solids phase (Table 1). To shape the geopolymer mass into the form of pots, the paste was poured into half volume of a plastic container. A second container (1/3 of the volume of the first one) was loaded with sand and placed into a form filled with mass. The form was set for 30 seconds on a shaker to eliminate air bubbles from the geopolymer mass. Geopolymer pots were heated in the laboratory dryer (Chemland) at 65 °C for 24 h, and then cured for 28 days at ambient conditions. After, the geopolymer neutralisation process was carried out in a hydrochloric acid solution. The effect of 0.1 M, 0.5 M, 1 M, and 8 M HCl was analysed. One-step HCl treatment versus two steps of HCl treatment (24 h each step) was also compared. The pots were washed in distilled water (24 h each rising), and the pH measurement was carried out using the Piccolo® plus pocket pH-meter, with an integrated electrode amplifier.

### 2.3. Geopolymer Properties Characterisation

Sample imaging was performed by a MOTIC SMZ-168 stereo microscope (Motic, Xiamen, China). The true density of the geopolymer materials was determined using a gas pycnometer, with a chamber volume of 20 cubic centimetres (Pycnomatic ATC), and 99.999% helium as the measurement gas. Open and total porosity was determined according to PN-EN 993-1:2019-01 using the Archimedes method (hydrostatic weighing with density determination set, Radwag). The closed porosity of the material was calculated based on the difference between total porosity and open porosity: $\%\;Total\;Porosity=\left(1-\frac{Bulk\;density}{True\;density}\right)\times 100$ [31]. The compressive strength of the geopolymer samples was determined using a rebound Schmidt hammer (Proceq, Schwerzenbach, Switzerland). The tests were carried out for samples before and after the neutralisation process. The impact energy was 0.735 Nm (0.075 kGm). The morphology of the materials was analysed with scanning electron microscopy JEOL-JSM-820 (JEOL, Tokyo, Japan). The metakaolin particles were dried to a constant mass, stuck on the coal tape, and coated with a thin layer of gold using the JEOL JEE-4X vacuum evaporator (JEOL, Tokyo, Japan). to ensure good conductivity of the samples. The microanalysis of the chemical composition was performed by SEM coupled with the energy dispersive spectroscopy (EDS) model IXRF 500 (IXRF Systems Inc., Austin, TX, USA). Spectroscopic analyses of the geopolymers were performed using the FT-Raman Nicolet NXR 9650 device (Thermo Scientific, Waltham, MA, USA), equipped with the Nd:YAG3 + laser with a wavelength of 1064 nm and InGaAs detector (Thermo Scientific, Waltham, MA, USA). For each sample, a minimum of 10 spots with a size of 50 µm was chosen using an integrated color video camera. Band identification was performed against spectral libraries and published results.

### 2.4. Geopolymer Pot Characterisation

Seeds of spring wheat, var. Almari, were germinated and grown for ten days in pots filled with small pieces of cellulose pats. The plant’s morphology was compared for plants grown in geopolymer pots vs plastic pots. Ion adsorption and desorption properties of the geopolymer foam pots were tested. The pots were immersed for 24 h in the mineral solution described above. Ten subsequent rinses with distilled water were performed. At each step, pots were filled with 50 mL distilled water and after 24 h, the leaches were analysed spectrophotometrically (Synergy 2 Multi-Mode Plate Reader, BioTek Instruments, Winooski, VT, USA). The procedures were carried out in accordance with the manufacturer’s instructions using commercial kits ZW 535550 (Slandi Ltd. Warsaw, Poland), 1-403-B125, 1-440-0080 (BioMaxima S.A., Lublin, Poland) for nitrogen, phosphorus, and potassium analysis, respectively, and standards for their content quantification (Química Clínica Aplicada S.A.).

## 3. Results and Discussion

### 3.1. Characterisation of Geopolymer Foams

**Figure 1:**
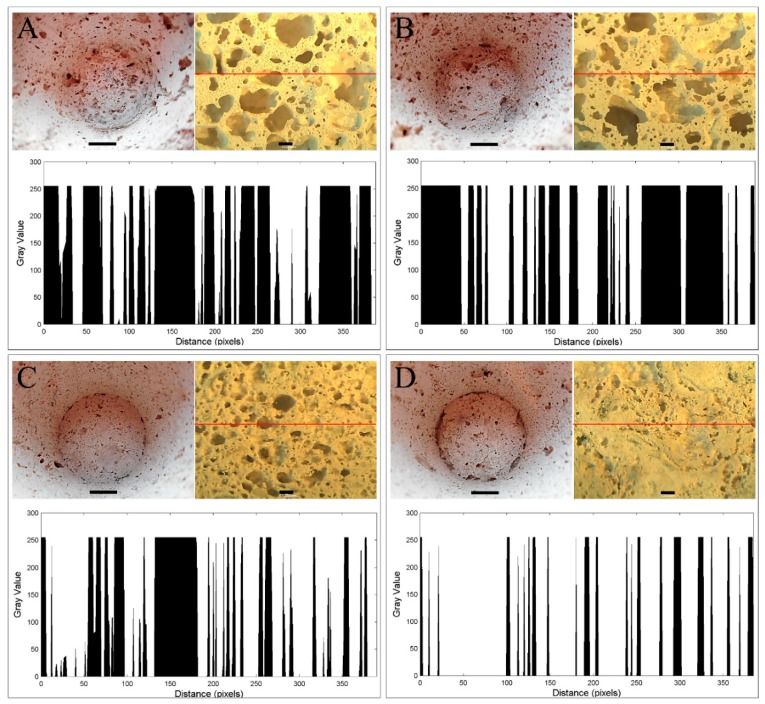
Macro- and microstructure, size and distribution of the pores in geopolymer pots G (**A**), GM (**B**), GC (**C**), and GCM (**D**). A marker of 1 cm was set for the inner surface of the pots (volume 100 mL). Marker of 1 mm was set for light microscopy photos. Size and distribution of the pores were measured using the plot profile analysis in the ImageJ 1.52a software (NIH, Bethesda, MD, USA) as a function of pixels number with the defined grey value. The ImageJ analysis was performed along the red lines marking the counterpart light microscopy photos.

**Figure 2:**
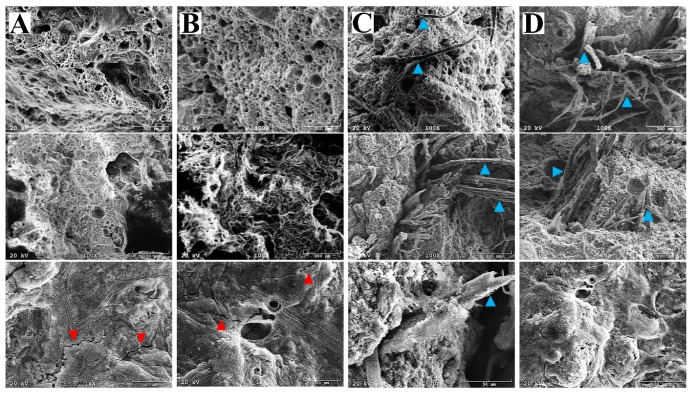
Representative microstructure of the basic geopolymer foam G (columns 1(**A**) and 1(**B**)), and the organic-geopolymers hybrid foams GC (columns 1(**C**) and 1(**D**)) before (1(**A**), 1(**C**)) and after (1(**B**), 1(**D**)) the two-step neutralisation procedure. In the structure of G, and GM samples, small cracks are marked with red arrows, and cellulose fibres fraction with blue arrows.

Porous materials are classified into several types by the size of the pores in the structure: Macroporous (pore diameters greater than 50 nm); mesoporous (2–50 nm); microporous (less than 2 nm) [44]. Figure 1 shows the pore size distribution and their morphological features in the geopolymers prepared according to different protocols. The porous structures of the produced geopolymers were heterogeneous and pores with different sizes were randomly distributed in the foams. In all geopolymer foam pots, macropores were in the size range of 0.2–4 mm (Figure 1, Table 2). The basic geopolymer foam (G) had the highest number of macropores, categorised by their size into the first class (4–2.5 mm), and a significantly increased number of pores from the fourth class (<0.2 mm), when compared to the other types of geopolymers. The pots GM had a reduced number of pores within most of the classes (Table 2). However, it resulted mainly from the coalescence of the pores and predomination of big pores in the structure (Figure 1B). Compositing the geopolymer foam with cellulose (GC) resulted in a dramatically reduced pore number in most of the classes, so the effect of the reinforcement was valid. However, similar to GM, the GC geopolymers improved the pore number with size in the range of 0.2–0.9 mm. The lowest number and the smallest size of pores characterised the surface of the organic-geopolymer hybrid foams with the addition of mineral solution (GCM). Despite such differences in pore size and number, the percentage of the total surface porosity was in most cases similar (approximately 56%), with the exception of GCM pots for which porosity was significantly reduced (28%). SEM images (Figure 2) showed the G samples also had the largest amount of pores in the scale range of 20–200 µm, whereas the minimum amount of pores occurred in the organic-geopolymer hybrid foams (GC). The geopolymer gel matrix presented strong bonding with cellulose fibres. Although they appeared to exhibit a random directional arrangement, a more or less homogenous distribution was observed at the microscale. In contrast, in the structure of the G sample, small cracks were visible.

The microscopic analysis was confirmed by measurement of the porosity by the hydrostatic method (Table 3). The results indicated that the pores constituted more than half of the material volume. The open porosity was dominant regardless of the type of sample. The addition of cellulose fibres and mineral solution reduced the closed, open, and total porosity of foamed geopolymer pots, indicating that the initial combination of components can control the porous structure of the material, the development of the surface of the channel system, and the density of the material. 

A coalescence of macropores was observed in the basic geopolymer foam pots with and without the addition of mineral solution (G, GM) as irregularities in pores shape (Figure 1). The pore circularity (pore shape factor) in both cases was approximately 0.44 (Table 2). Reinforcement of the geopolymers with cellulose fibres reduced the coalescence of pores and improved the pores’ circularity to 0.77. In the foaming process, the density of components, and thus the viscosity of the liquid, play a significant role in the pore’s formation and their distribution. Increasing the viscosity of the liquid foaming reduces the gases removal, and when the membrane between two bubbles is broken because of insufficient stabilisation, the bubbles can merge into larger bubbles with non-spherical shapes. The density of metakaolin was 2.6 g cm^−3^, while the addition of cellulose, with an initial density of 1.5 g cm^−3^ (apparent density 0.060 g cm^−3^), reduced the viscosity and then the coalescence. This was additionally supported by the final density of the foams (Table 3); the reinforcement with cellulose reduced the true density as determined by the pycnometric method (the mass of a material divided by its volume, excluding open and closed pores). While the basic geopolymer foam pots (G), with and without the addition of mineral solution (G, GM), had a similar density value of approximately 2.5 g cm^−3^; the addition of cellulose fibres to the geopolymer matrix (GC, GCM) resulted in a reduction in its density to about 2.3 g cm^−3^. Furthermore, an excess of the blowing agent can induce coalescence. Hydrogen peroxide reacts, evolving oxygen gas, which is entrapped in the geopolymer paste. Equations: H_2_O_2_ + OH^−^ ⟶ HO_2_^−^ + H_2_O, and HO_2_^−^ + H_2_O_2_ ⟶ H_2_O + O_2_ + OH^−^, represent the reactions during the foam formation [45]. It has been suggested that if the amount of H_2_O_2_ is too high, the pores were not well distributed because of the bubble’s buoyancy [46]. Indeed, the effect of random distribution of the pores was observed for G and GM foams. In this context, comparing the porous structure of the GC and GCM foams, it can be concluded that the cellulose addition changed the gaseous/liquid/solid ratio. Cellulose in the geopolymer paste can be partially depolymerised. Products of the depolymerisation can be oxidised along with the consumption of excess H_2_O_2_, thus changing the O_2_ and H_2_O ratio and resulting in a different foam porosity. 

**Figure 3:**
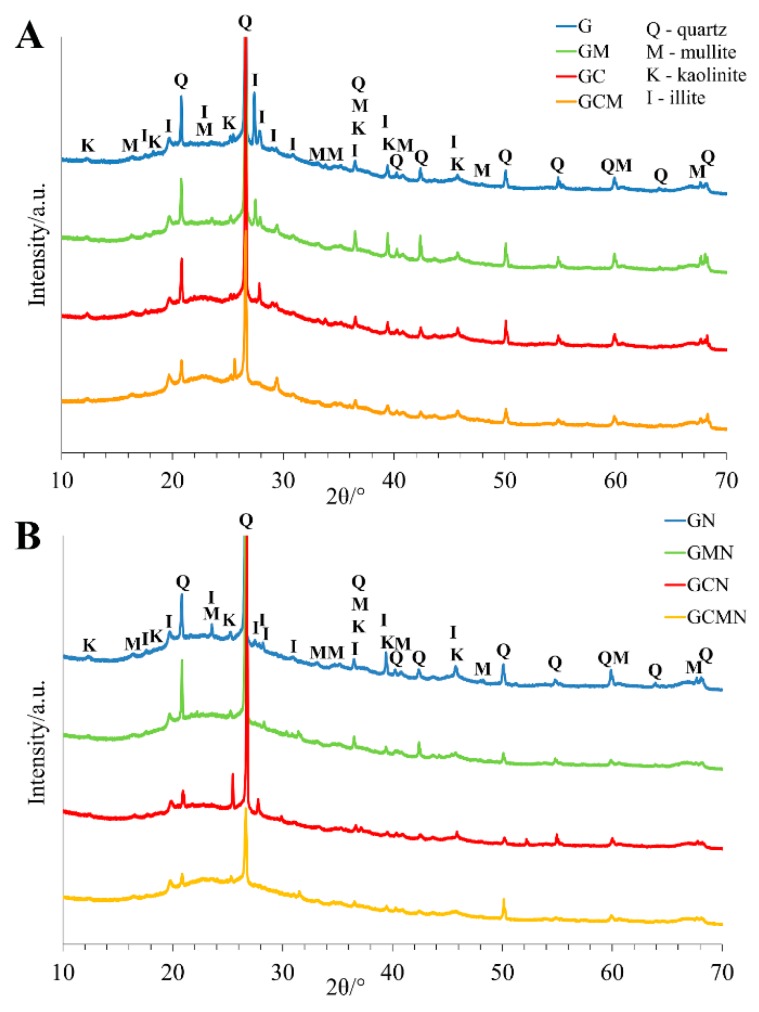
The XRD patterns of the geopolymer foam (G, GN), the geopolymer foam with the addition of mineral solution (GM, GMN), and the organic-geopolymers hybrid foams with and without the addition of mineral solution (GC, GCN, and GCM, GCMN, respectively) before (**A**) and after (**B**) the two-step neutralisation procedure.

The X-ray diffractograms of the geopolymer foams (Figure 3) showed the presence of all the crystalline phases initially encountered in the metakaolins [47] i.e., the presence of quartz (SiO_2_), mullite (3Al_2_O_3_2SiO_2_/2Al_2_O_3_SiO_2_), kaolinite (Al_2_Si_2_O_5_(OH), and illite (K,H_3_O)(Al,Mg,Fe)_2_(Si,Al)_4_O_10_[(OH)_2_,(H_2_O)]. However, the comparison between metakaolin [47] and geopolymer foams (Figure 3) revealed certain changes with regard to the halo peak representing the amorphous phase. The halo peak with 2θ between 18° and 32°, for basic metakaolin, had a higher intensity and was extended to 40° for geopolymers. An amorphous hump could be due to the presence of amorphous glassy materials, where the diffraction crystals were those of the original mullite and quartz (metakaolin KM 60: 50%–55% SiO_2_).

### 3.2. Neutralisation of Geopolymers after Alkaline Activation

Materials can be designated to agricultural applications, provided they have neutral properties. Amongst others, one of the key factors is pH. The desirable pH in the range of 6.0–7.5 is acceptable for optimum plant growth, whereas pH > 8.3 and pH < 4.5 is too alkaline and too acid, respectively. First, plants in the pots made from materials for which pH is beyond the optimal range could be directly damaged (Figure S1 in Supplementary Data). Second, in highly alkaline soil, macronutrients (N) and most micronutrients (Fe, Mn, Cu, Zn) become less available for the plants, while in highly acidic soil a deficiency of N, P, K, S, Ca, Mg and Mo may occur together with a toxic excess of Al, Fe, and Mn [48]. In both cases, the growth of the plants is reduced due to insufficient soil conditions. On the other hand, the geopolymerisation process requires the dissolution of the starting material in a high pH (alkaline) solution, and thus pH values of fresh geopolymer pastes are usually 11.2–13.2 [34]. The pH of geopolymer pots can be lowered with acidic agents; however, we showed that acidification conditions require careful optimisation.

Table 4 and Table 5 present the results of the two tests carried out in order to optimise the pH of the alkali-geopolymer samples with HCl solution. In the first one, the most efficient concentration of HCl and the dynamics of pH changes in the following days after the neutralisation process were determined. The geopolymer foam pots (G), with initial pH = 11.18, were soaked in 0.1 M, 0.5 M, 1 M, or 8 M HCl solution (pot:solution; 1:10; v/v) for 24 h. After this period, the pots were transferred to distilled water (pot:water; 1:10; v/v). The pH was measured after 24 h (day 1st). On each of the following days (2nd–33rd), the post-rinsing water was replaced with clean distilled water, and pH of the leachates was measured after the next 24 h. The results indicated that 0.5 M HCl was the most suitable solution since the optimal pH (6.35) had already been reached after 5 days (4 rinses), and its value was stable in the following days of rinsing (Table 4). The effect of neutralisation with 0.1 M HCl solution was minor, and the final pH value stabilised at the level of 9.36. In contrast, 1 M and 8 M HCl solutions were too acidic, and the pH decreased to very low values. Its recovery to pH ~7.0 took 11 days (7 rinses) after neutralisation in 1 M HCl, and even 33 days were not enough to reach the optimal pH of the leachates after neutralisation with 8 M HCl solution. In the second test, the basic geopolymer foam pots (G), the geopolymer foam pots with the addition of mineral solution (GM), and the organic-geopolymer hybrid foams, with and without the addition of mineral solution (GC and GMC, respectively) were compared in a two-step neutralisation procedure. The samples were treated twice with 0.5 M HCl solution, and the two treatments were separated by rinsing with distilled water. The addition of inorganic minerals or organic cellulose slightly lowered the initial pH of geopolymer foams (Table 5). The neutralisation was effective in the case of all types of geopolymer foams with the first step reducing the pH to values of about 8.5, and the second to the optimal values of about 7.0. Taking into consideration the number of steps (two HCL treatments and one water rinsing), the time required for neutralisation (three days), and the volume of liquid used (3 litres per pot); the second neutralisation protocol was much more effective and less expensive than the first one (one step of HCl treatment and minimum 4 rinsings, minimum 5 days, and a minimum 5 litres of liquids).

The geopolymers did not show significant changes in appearance and organoleptic properties after immersion in acid solution. However, both the neutralisation process and the reinforcement of geopolymers with cellulose eliminated any efflorescence—white salt deposits on or near the surface of pots—which can occur after 90 days of aging (Figure S2 in Supplementary Data). The micro-structure of both the basic geopolymer foam G, and the organic-geopolymer hybrid foam GC, were not disturbed after the neutralisation process (Figure 2).

The strength of geopolymer samples, determined using a Schmidt hammer, was slightly lower when G foams were previously neutralised (Table 6). Reinforcement of the geopolymer GC with cellulose has already improved the strength of samples before the neutralisation process, and clearly prevented the reduction of mechanical properties after neutralisation of GC foam. After acidic treatment, the strength values of the GC and GCM geopolymers were higher, in a range of 16–20%, when compared to the basic G geopolymer.

First, it can be the effect of a lower susceptibility of cellulose to the acidic agent when compared to the geopolymers. Second, the total volume of pores correlates with the density and the strength of the geopolymer foam. It is generally agreed that large pores or voids weaken the structure more than small pores in the geopolymer material, which is in agreement with the larger pore size in the G and GM foams than in the GC and GCM foams. Additionally, the coalescence of macropores observed in the G and GM foams can cause a decrease in the flexural strength found in these samples. Third, the presence of small cracks in the G foam (but not in GC) exposed to the acidic agent, can further increase the susceptibility of the basic geopolymer foams G. Fourth, the strength of the geopolymer material depends on the matrix strength [46]. Uniform dispersion of cotton fibres in the slurry could result in an improvement of the consistency of the matrix as well as high wettability between the fibres and the paste during the geopolymerisation process. Additionally, the use of alkaline solutions cleans the surface of cellulose fibres from impurities and waxes, increases the surface roughness of the fibres and improves their adhesion. The increase of the adhesion at the matrix and cotton fibre interface improves their bonding strength. This permits the optimum operation of stress-transfer from the matrix to the cotton fibres and results in an improvement in the strength properties. In earlier studies, the addition of cotton fibres led to a reduction in compressive strength instead of an improvement, and it was attributed to a greater balling together of the fibres, as a result leaving voids in the matrix [49], which was not observed in our foams. Fifth, when cotton fibres are in excess, they can absorb too much water [50], and thus deny the geopolymers around the fibres sufficient water for geopolymerisation, and in turn, decrease the bonding strength between the fibre and the matrix. Therefore, one can conclude that the ratio of cellulose fibres and geopolymer paste was optimal in the GC foams. Sixth, EDS analysis, performed to observe the composition of the geopolymers, showed that oxygen, sodium, aluminium, and silica are the major components, thus the formed geopolymers mostly consist of the phases containing Na–Si–Al in the bulk region, suggesting the formation of a silicate-activated gel by polymerisation throughout the inter-particles volume (Table 7). However, treatment of a geopolymer with a strong acid may cause the breakage of the Si–O–Al bonds, and the greater the number of Si–OH and Al–OH groups in geopolymers, the greater the amount of silicic acid ions and dimers. Furthermore, the liberation of silicic acid from the samples, the replacement of Na and K cations by hydrogen or hydronium ions, and dealumination of the geopolymers can occur [51]. The dealumination process leads to a mass loss of the geopolymer materials, however, neither mass decrease, nor microstructure changes were observed in the G geopolymer. Possibly, depolymerisation of geopolymers was followed by condensation of polymeric ions enriched with silicon, then either amorphous polymers or zeolites precipitated in geopolymers [51]. Indeed, dealumination of the geopolymers and then the condensation process caused an increase of the Si:Al ratio. For G samples, the Si:Al ratio before neutralisation was 11.94:12.06, while after neutralisation the Si:Al was 29.94:3.24 (Table 7). Further, the higher and wider halo peak with 2θ between 18° and 40° was observed for the neutralised G geopolymer in comparison to the untreated G geopolymer, suggesting rather an amorphous phase, than zeolites. It was shown earlier, that the reactions generating amorphous polymers, ensure good durability of the geopolymer, while precipitation of zeolite was associated with the loss of strength [51]. It can explain that the basic G geopolymer did not change its weight and only had about 11% strength reduction. Although the neutralisation drastically reduced the Na, and Al content in G foams (300% and 37%, respectively), the reduction for GC samples was to a lesser extent (58%, and 15%, respectively). It suggests that chemical interaction between the organic and inorganic polymeric chains can prevent modification of the geopolymer structure in acidic conditions. Seventh, the XRD data of geopolymer foams showed a significant change in percentage of mullite and illite after the neutralisation process, compared to the relative content of mullite and illite in the geopolymers before acidic treatment (Table 8). Both mullite and illite are rich in aluminium (Al > Si), so their reduction can be correlated with the dealumination process demonstrated in the EDS analysis. In particular, the decrease of the mullite and illite was observed for the basic geopolymer with, and without, the addition of mineral solution (G and GM, respectively). For geopolymers enriched with organic fibres GC, a percentage reduction was found to a small extent, while in the case of geopolymers enriched with organic fibres and mineral solution GCM, even higher percentages of mullite and illite were found.

Altogether, this implies the feasibility of using cotton fibres to mitigate brittle failure in geopolymers and their protection from the negative effect of acidic agents (neutralisation).

### 3.3. Geopolymer Foam Pots for Plant Cultivation

**Figure 4:**
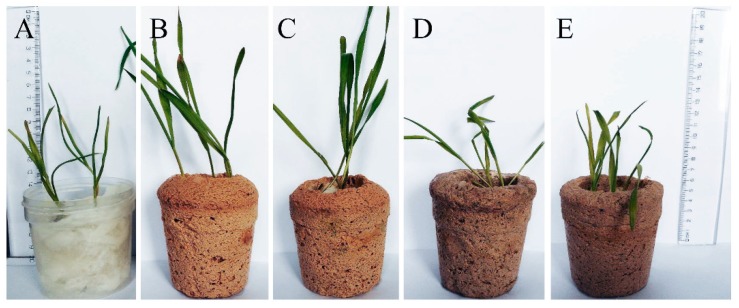
Representative example of the growth of wheat plants in: Plastic pot (**A**), the G geopolymer foam (**B**), the GM geopolymer foam with the addition of mineral solution (**C**), the GC and GCM organic-geopolymers hybrid foams with, and without the addition of mineral solution, respectively (**D**,**E**) subjected to neutralisation process.

The requirements for application of the geopolymer pots in plant cultivation are not only the properties that allow them to be used as an alternative to plastic pots. Better plant growth in the geopolymer pots was also expected due to the higher air permeability of the pots, and their mineral composition. Therefore, germination and growth of spring wheat in geopolymer pots was tested versus plant growth in a plastic pot (Figure 4). Much faster growth, greener leaves, and thicker leaf lamina, especially in the G and GM pots, was observed. Slightly slower, but still significantly improved plant growth was found in the GC and GCM pots in comparison to plastic pots. It could be associated with the different structure of the pots, with and without cellulose fibres (different porosity). Most surprisingly, however, was that the morphology of the plants changed. Plants had shorter but much thicker leaves, and plant biomass was greater than for plastic-grown plants. Such plant behavior indicates that the growth conditions were good. In contrast, slightly longer, but much thinner leaves of plants grown in the plastic boxes indicated less biomass accumulation as a result of less suitable conditions for plant growth. It also indicates the effect of released ions from the pot structure (higher ions availability), despite the relatively simple elemental composition of the geopolymer. Furthermore, one can conclude a high success of the neutralisation process when improved growth of the plant in neutralised pots was compared to drastic inhibition of plant growth—or even plant death at the beginning of germination—in pots before neutralisation process (Figure S1 in Supplementary Data).

**Figure 5:**
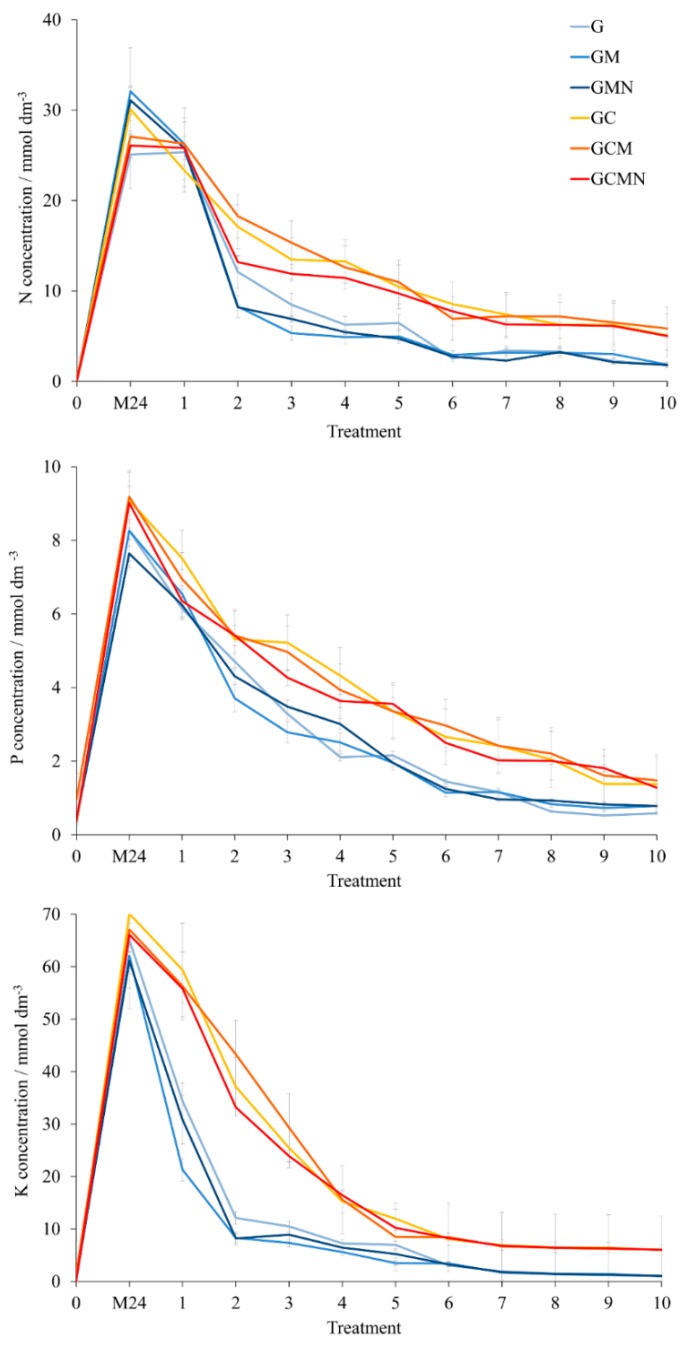
Nitrogen, phosphorus and potassium concentration in a solution after rinse untreated pots (0), pots soaked 24 h with the mineral solution (M24), and subsequent ten rinses of M24 pots. G-basic geopolymer, GM-G with the addition of mineral solution during geopolymerisation process, GMN–GM neutralised after alkali-activation, GC–geopolymer reinforced with cellulose, GCM–GC with the addition of mineral solution during geopolymerisation process, GCMN–GCM neutralised after alkali-activation.

Taking into consideration the simple chemical composition of the pots, which does not guarantee plant nutrition during whole vegetation, and the fact that the soil in pots loses nutritional properties with time due to nutrient uptake by plants, it is necessary to fertilise plants during the growing season. Therefore, the ion adsorption and desorption in the geopolymer foam were tested. The pots were soaked for 24 h in the solution of standard fertiliser, and 10 subsequent rinses were performed, which was the equivalent of watering the plants in growing conditions (Figure 5). The results indicate that the initial content of mineral compounds in all pots was near zero, with the exception of phosphorus in the GM and GCM pots, which were treated with the mineral solution during the geopolymerisation process. However, in this case, the neutralisation process caused the loss of minerals, since the N, P, and K content was below the detection level in the GMN and GCMN pots. After the pots were soaked for 24 h with the mineral solution, it was found that a higher content of mineral ingredients was absorbed in the pots reinforced with cellulose (GC, GCM, GCMN), when compared to basic geopolymers (G, GM, GMN). The cellulose in geopolymers also resulted in a slower desorption of minerals during subsequent rinsing, in contrast to the more rapid decrease of individual components in pots G, GM, and GMN. This effect could be associated with a lower number of large pores in the presence of cellulose fibres in pots, thus a more stable pore filling, as well as better protection of internal surface interactions, including non-ionic adsorption into the internal structure of the geopolymer, and ion-exchange adsorption through electrostatic interaction.

However, it is important to note that plants in the geopolymer pots needed more water than in plastic pots. The geopolymer pots easily evaporate the excess water, due to the fact that water can be transported from the internal pot walls to the external walls through capillaries in the material. This is unsuitable due to economic and environmental reasons, therefore additional treatments of the outside wall of the pots (i.e., a layer of paint or impregnation) should be considered to obtain fully functional geopolymer pots for industrial production and cultivation of a large number of plants in greenhouse conditions.

## 4. Conclusions

Low-cost geopolymer foam materials could be produced from metakaolin and sand, using an alkali activation process (NaOH) and foaming agents (H_2_O_2_). The excellent potential of a two-stage neutralisation method was demonstrated, which allows the use of geopolymers for the production of pots with approximately pH 7, and thus, for plant cultivation. Geopolymers can be reinforced with cellulose fibres, which protect the geopolymer structure during the neutralisation process (preventing dealumination) and improves the chemical, physical, and mechanical properties of geopolymer foams. 

Great adsorption/desorption properties allow soaking the geopolymer foam pots in a mineral solution for a long-term fertilisation effect. Geopolymer foams reinforced with cellulose fibres were found to distribute the macroelements and supply them to plants in a more uniform way as well as to prevent them being washed-out during watering. The positive impact on plant growth was observed when they were planted in geopolymer foam pots, as they had greater growth compared to plants that had been grown in plastic containers. In conclusion, bio-geopolymer pots are a suitable alternative to plastic pots.

## Figures and Tables

**Table 1:** Steps of geopolymer production, and composition of geopolymer materials.

Sample	Geopolymer Component			Foaming Component		Additives		Liquid: Solid **
	Metakaolin: Sand 9:1 (wt %)	Water Glass: NaOH 2.5:1 (wt %)	Liquid: Solid *	H_2_O_2_ (wt %)	Al (wt %)	Mineral Solution (wt %)	Cellulose Fibres (wt %)	
G	58.84	38.40	0.65	2.14	0.62	0.00	0.00	0.68
Metakaolin + sand »»» NaOH + water glass »»» H_2_O_2_ + Al »»» G								
GM	58.84	34.87	0.59	2.14	0.62	3.53	0.00	0.68
Metakaolin + sand »»» NaOH + water glass »»» mineral solution »»» H_2_O_2_ + Al »»» GM								
GC	51.38	43.41	0.84	1.97	0.57	0.00	2.67	0.82
Metakaolin + sand »»» cellulose »»» NaOH + water glass »»» H_2_O_2_ + Al »»» GC								
GCM	51.38	40.17	0.78	1.97	0.57	3.24	2.67	0.82
Metakaolin + sand »»» cellulose »»» NaOH + water glass »»» mineral solution »»» H_2_O_2_ + Al »»» GCM								

* Geopolymer components i.e., meatakaolin and sand: Alkali activator, ** all components.

**Table 2:** The frequency of pores categorised by their size into four groups, the relative surface area of the pores and their circularity. All parameters were measured for 1 mm^2^ surface of the geopolymer pots G, GM, GC, and GCM, using the ImageJ 1.52a software (NIH, USA). Standard deviation was less than 10% of the mean values (n = 10).

Sample	Number of the Pores with Defined Size				Porosity (%)	Circularity
	4.0–2.5	2.4–1.0	0.9–0.2	<0.2		
	(mm)					
G	7.05	8.98	27.57	769.45	56.847	0.438
GM	3.85	5.13	41.04	439.87	57.425	0.442
GC	1.92	7.69	112.85	336.64	54.436	0.770
GCM	0.00	3.21	85.92	160.30	28.428	0.219

**Table 3:** The porosity of geopolymer foam pots and their density.

Sample	Closed Porosity (%)	Open Porosity (%)	Total Porosity (%)	True Density (g cm^−3^)
G	21.98 ± 2.28	45.21 ± 2.42	67.19 ± 2.11	2.524 ± 0.008
GM	20.70 ± 3.61	43.50 ± 3.90	64.20 ± 6.13	2.503 ± 0.012
GC	20.10 ± 4.29	41.87 ± 2.98	61.97 ± 3.74	2.314 ± 0.001
GCM	19.43 ± 5.23	33.18 ± 6.90	52.61 ± 1.68	2.319 ± 0.001

**Table 4:** pH changes of basic geopolymer foam pots in the following days after the one-step neutralisation process performed in 0.1 M, 0.5 M, 1 M, and 8 M HCl. Color intensity is an indicator of alkalinity (blue) and acidity (red). Standard deviation was less than 10% of the mean values (n = 3).

HCl	Following Days after the Neutralisation Process											
	1st	2nd	4th	5th	7th	8th	11th	12th	28th	29th	32nd	33rd
0.1 M	2.79	6.94	7.75	8.52	8.83	9.35	9.33	9.38	9.27	9.4	9.26	9.36
0.5 M	0.55	2.67	4.39	6.35	6.35	6.36	6.38	6.38	6.38	6.37	6.36	6.32
1 M	0.12	1.05	3.7	4.8	5.59	5.68	7.46	7.58	7.05	7	6.9	6.96
8 M	0.13	0.15	1.91	2.84	3.65	4.2	4.67	4.2	5.75	5.7	5.68	5.66

**Table 5:** pH changes of the geopolymer foam pots (G), the geopolymer foam pots with the addition of mineral solution (GM), and the organic-geopolymer hybrid foams with and without the addition of mineral solution (GC and GCM, respectively) during the two-step neutralisation procedure in 0.5 M HCl. Colour intensity is an indicator of alkalinity (blue). Values represent mean values ± standard deviation (n = 3).

Sample	Initial pH	pH after 1st Neutralisation	pH after 2nd Neutralisation
G	11.18 ± 0.11	8.06 ± 0.13	6.67 ± 0.21
GM	10.25 ± 0.05	8.61 ± 0.95	7.06 ± 0.05
GC	10.59 ± 0.21	8.89 ± 0.27	7.76 ± 0.28
GCM	10.24 ± 0.11	8.62 ± 0.62	7.1 ± 0.08

**Table 6:** Strength of the geopolymer foam pots (G), the geopolymer foam pots with the addition of mineral solution (GM), and the organic-geopolymers hybrid foams with, and without the addition of mineral solution (GC, and GCM, respectively) before, and after the two-step neutralisation procedure. Strength was determined using a Schmidt hammer. Values represent mean values ± standard deviation (n = 10).

Sample	Strength before Neutralisation (N mm^−2^)	Strength after Neutralisation (N mm^−2^)
G	17.0 ± 1.0	15.0 ± 0.8
GM	16.7 ± 0.6	15.7 ± 1.2
GC	18.3 ± 1.5	17.5 ± 0.6
GCM	18.0 ± 1.7	18.0 ± 1.7

**Table 7:** Results of EDS analysis of the geopolymer foam pots (G), and the organic-geopolymers hybrid foams (GC) before and after the two-step neutralisation procedure. Standard deviation was less than 10% of the mean values (n = 5).

Element	Intensity (c s^−1^)	Content		Error 2-sig
		Atomic (%)	Weight (%)	
**G** **before Neutralisation**				
O K_α_	0.37	57.05	45.43	13.56
Na K_α_	0.45	18.95	21.68	5.92
Al K_α_	0.46	12.06	16.20	4.47
Si K_α_	0.47	11.94	16.69	4.41
**GN after Neutralisation**				
O K_α_	16.65	66.19	52.90	2.40
Na K_α_	0.63	0.64	0.73	0.22
Al K_α_	7.38	3.24	4.36	0.32
Si K_α_	76.96	29.94	42.01	0.79
**GC before Neutralisation**				
O K_α_	2.85	59.06	46.00	6.01
Na K_α_	1.03	5.12	5.74	1.14
Al K_α_	5.42	13.05	17.14	1.45
Si K_α_	8.99	22.77	31.13	1.94
**GCN after Neutralisation**				
O K_α_	8.67	67.09	54.12	3.50
Na K_α_	0.41	0.88	1.02	0.33
Al K_α_	9.55	8.88	12.08	0.74
Si K_α_	25.18	23.15	32.79	1.11

**Table 8:** Percentage of quartz (SiO_2_), mullite (3Al_2_O_3_2SiO_2_/2Al_2_O_3_SiO_2_), kaolinite (Al_2_Si_2_O_5_(OH), and illite (K, H_3_O)(Al, Mg, Fe)_2_(Si, Al)_4_O_10_[(OH)_2_, (H_2_O)] in geopolymer foam pots before, and after the two-step neutralisation procedure.

Sample	Before Neutralisation				After Neutralisation			
	Q	M	K	I	Q	M	K	I
G	14.8	4.1	15.4	65.7	16.2	2	34.2	47.6
GM	15.5	4.2	21.3	59.1	22.3	1	54.1	22.7
GC	15.7	4.7	22.6	57	11.1	4.2	33.4	51.3
GCM	13.2	4.4	32.5	49.9	10.2	5.1	22.3	62.4

## Supplementary Materials

The following are available online at https://www.mdpi.com/1996-1944/12/18/2999/s1, Figure S1: 7 day old plants in the plastic pots as well as in geopolymer foam pots before the neutralisation procedure, Figure S2: An efflorescence (white salt deposits) on or near the surface of pots, which were not subjected to the neutralisation process. Presented pots after 90 days of aging at ambient temperature and humidity.
