# Supplementary material for: Optimal Design of pH-neutral Geopolymer Foams for Their Use in Ecological Plant Cultivation Systems
Source: Materials (Basel). 2019 Sep 16;12(18):2999. doi: 10.3390/ma12182999 (PMC6766294; doi:10.3390/ma12182999)
Supplement: Supplementary file 1 [file materials-12-02999-s001.pdf]

Article

Optimal Design of pH-neutral Geopolymer Foams for Their Use in Ecological Plant Cultivation Systems

Magdalena Szechyńska-Hebda [1,2,*], Joanna Marczyk [3], Celina Ziejewska [3], Natalia Hordyńska [1], Janusz Mikuła [3] and Marek Hebda [3,*]

[1] The Franciszek Górski Institute of Plant Physiology Polish Academy of Sciences, Niezapominajek 21, 30-239, Cracow, Poland; n.hordynska@ifr-pan.edu.pl

[2] The Plant Breeding and Acclimatization Institute - National Research Institute, Radzików, 05-870 Błonie, Poland

[3] Institute of Materials Engineering, Cracow University of Technology, Warszawska 24, 31-155 Cracow, Poland; jmarczyk94@gmail.com (J.M.); celina.ziejewska@gmail.com (C.Z.); jamikula@pk.edu.pl (J.M.)

* Correspondence: m.szechynska@ifr-pan.edu.pl (M.S.-H.); mhebda@pk.edu.pl (M.H.); Tel.: +48-12-4251833 (M.S.-H.); +48-12-6283423 (M.H.)

Received: 18 August 2019; Accepted: 13 September 2019; Published: date

Supplementary Materials: Figure S1 and Figure S2.

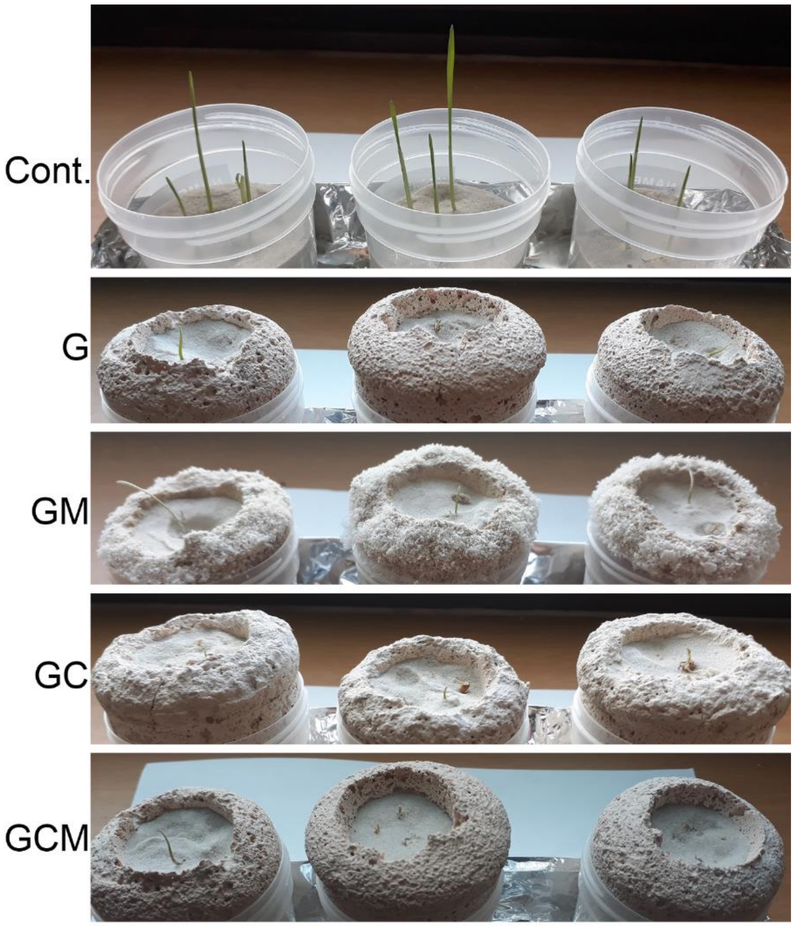

Figure S1. 7-day old plants in the plastic pots as well as in geopolymer foam pots before neutralisation procedure. Plastic pots (Contr.); basic geopolymer foam pots (G), the geopolymer foam pots with the addition of mineral solution (GM), and the organic-geopolymer hybrid foams with and without the addition of mineral solution (GC and GCM, respectively). Plants in the pots made from materials for which pH is beyond the optimal range (pH approx. 12) could be directly damaged showing drastic inhibition of growth or even plant death at the beginning of germination.

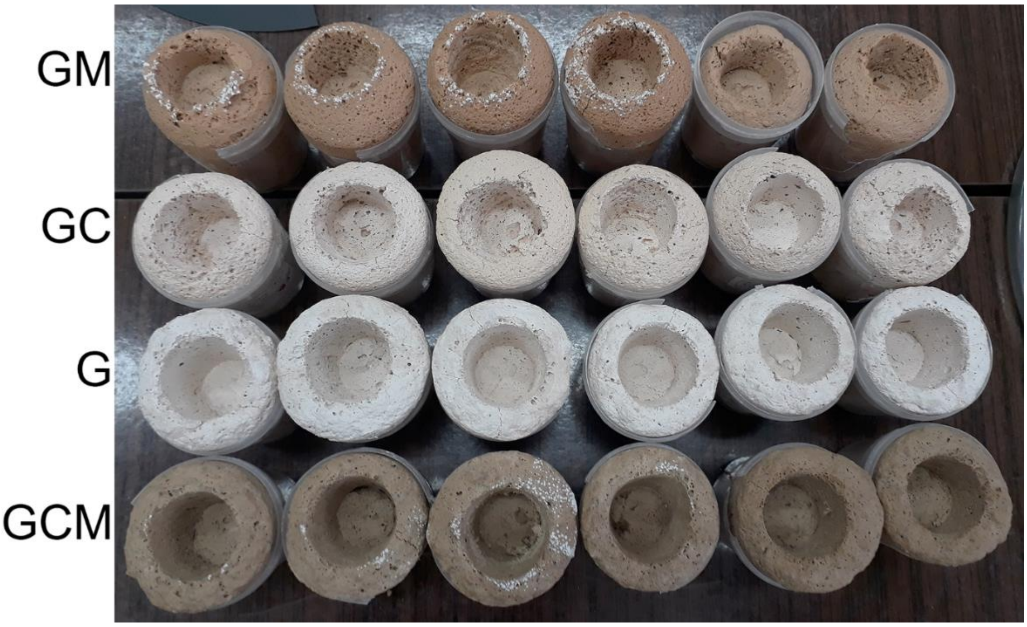

Figure S2. An efflorescence (white salt deposits) on or near the surface of pots, which were not subjected to neutralization process. Presented pots after 90 days of aging at ambient temperature and humidity. .
